# Unrecognized and Unheard: Experiences of Black Mothers Navigating the “Good NICU Mother” Ideal Across the Preterm Birth Continuum

**DOI:** 10.21203/rs.3.rs-9371418/v1

**Published:** 2026-05-19

**Authors:** Karen Warren, Robin Dail, Tisha Felder, Nansi Boghossian, Robin Dawson

**Affiliations:** University of South Carolina; University of South Carolina; University of South Carolina; University of South Carolina; University of South Carolina

**Keywords:** Respectful maternity care, Preterm birth, Neonatal intensive care, Black maternal health, Racial inequity, Qualitative research, Maternal identity, Neonatal transport, Structural racism, Health disparities

## Abstract

**Background:**

Black mothers who experience preterm birth and neonatal intensive care unit (NICU) hospitalization face intersecting structural and institutional challenges that shape their early maternal experiences. While racial disparities in maternal and neonatal outcomes are well documented, less is known about how racialized narratives of Black motherhood are enacted across everyday perinatal care interactions. Grounded in the principles of respectful maternity care (RMC), this study examines how Black mothers navigate birth, neonatal transport, and NICU hospitalization, and how institutional expectations of the “good NICU mother” shape maternal identity and legitimacy within these settings.

**Methods:**

This qualitative study used a reflexive thematic analysis of in-depth interviews with five Black mothers whose very preterm infants (24–28 weeks’ gestational age) required neonatal transport to a tertiary NICU in South Carolina. Interviews were conducted during or shortly after the infant’s NICU hospitalization and analyzed using Braun and Clarke’s six-phase approach. Findings were contextualized using an adapted National Institute on Minority Health and Health Disparities research framework.

**Results:**

Three phases of experience were identified: (1) preterm labor and initial contact with the healthcare system, (2) preterm birth at a referral hospital and neonatal transport, and (3) the postpartum period during NICU hospitalization. Across all three phases, participants described dismissal of their concerns, constrained communication, limited agency, and physical and emotional mistreatment — experiences that directly conflict with the core principles of RMC. The “good NICU mother” emerged as a regulatory standard — defined by constant bedside presence, emotional composure, breastmilk production, and fluency in medical terminology — that participants were evaluated against but structurally unable to meet. For Black mothers, these expectations intersected with racialized assumptions about maternal competence and responsibility, intensifying experiences of surveillance and scrutiny.

**Conclusions:**

Institutional systems of perinatal care function as sites where maternal legitimacy is interpreted and evaluated, often in ways that undermine RMC for Black mothers. Extending RMC principles beyond labor and delivery into neonatal intensive care settings is essential for achieving equitable, dignity-centered care. Structural reforms to visitation policies, documentation practices, and communication standards are needed across the full perinatal continuum.

## INTRODUCTION

The journey through pregnancy and childbirth represents the most significant physical and psychological changes a woman can experience [1, 2]. Becoming a mother is a fluid, evolving process influenced by the mother's personal experiences at all levels - individual, interpersonal, community, societal, and historical [3]. This transition can be challenging and even distressing for mothers who experience preterm birth (PTB) and have an infant hospitalized in the neonatal intensive care unit (NICU) [4]. Hospital policies and other demands and obligations (e.g., caring for older children, employment demands, financial constraints) can prevent mothers from being with their infants continuously. Limited presence can disrupt bonding and lead to adverse consequences for infants (e.g., separation anxiety, failure to thrive), as well as depression and anxiety for parents [5]. In fact, many mothers exhibit signs of psychological trauma due to a stressful birth experience, which is then compounded by prolonged separation and uncertain outcomes for their infant [6, 7]. Institutional factors, such as the stressful NICU environment, healthcare providers’ negative attitudes toward parental presence or participation in their infant’s care, and inadequate communication from providers to parents, further exacerbate these outcomes [8].

### The Impact of Racism and Implicit Bias in Healthcare

The intersections of social and structural drivers of health further impact mothers who experience PTB. Overt racism and implicit biases have been recognized as key drivers of persistent inequities in maternal and neonatal care [9, 10]. Historical myths of fundamental and biological differences- embedded in medical education and practice- positioned the belief that Black patients are less sensitive to pain and more biologically resilient, rationalizing differential treatment [11–13]. Although these beliefs are often framed as relics of the past, present day evidence continues to document the dismissal of Black patients’ symptoms, unequal pain management, delayed treatment, and disregarding of the bodily experiences [14–17]. These persistent and often invisible assumptions directly result in poorer outcomes (e.g., Black infants remain more than twice as likely to die in the first year of life compared to white infants [18]), reflecting enduring structural disparities across the prenatal period.

These inequities operate not only through discrete clinical decisions, but also through everyday interactions that shape whose knowledge is considered credible and whose concerns are deemed legitimate. When Black women’s bodily experiences are discounted, they are positioned as less authoritative narrators of their own pregnancies and births. Such institutionalized assumptions may extend beyond treatment decisions to influence how maternal competence, responsibility and worthiness are interpreted. In this way, racialized histories of credibility and suspicion become intertwined with contemporary expectations of what constitutes a “good mother”, shaping how maternal behavior is evaluated within clinical spaces, including NICU.

### Respectful Maternity Care and the NICU

The World Health Organization defines respectful maternity care (RMC) as care that maintains dignity, privacy, and confidentiality, ensures freedom from harm and mistreatment, and enables informed choice and continuous support during labor and birth [19]. Recognized as a fundamental human right, RMC has gained significant scholarly and policy attention as a framework for understanding and addressing disrespect and abuse in maternity care globally. However, existing RMC scholarship has focused predominantly on the intrapartum period, with comparatively little attention paid to how these principles extend into postnatal and neonatal care settings. For Black mothers whose infants require NICU hospitalization following preterm birth, the conditions that define RMC — dignity, freedom from mistreatment, and informed choice — do not end at delivery. The NICU represents a continuation of the maternity care continuum, and one in which racialized assumptions about maternal competence and worthiness may further undermine respectful care. Understanding how RMC is experienced — or denied — across this extended continuum is essential for addressing the structural and institutional forces that shape Black maternal health.

### Becoming a “good NICU mother

Understanding how motherhood becomes subject to evaluation requires attention to its social construction. Drawing on Judith Butler’s [20] theory of gender performativity, social identities are not fixed traits but are produced through repeated acts within regulatory frameworks. When gender is performed “correctly”, it is rewarded by societal approval; conversely, an individual can be punished if societal expectations related to gender are not met. Similarly, there exists a performativity of motherhood which constitutes more than merely giving birth. It is enacted and recognized through patterned behaviors, expectations, and institutional norms, and when performed “correctly”, it is rewarded by societal approval. Conversely, this construction can result in punitive repercussions when performed incorrectly [21]. Historical analyses demonstrate that women who deviated from dominant maternal norms faced profound social consequences. For example, narratives of once-unwed mothers reveal experiences of intense stigma, feeling so shamed by society that they surrendered their child for adoption [22]. Such accounts illustrate how maternal legitimacy has long been contingent upon conformity to prevailing social standards.

There is a “good mother” ideology, traditionally portrayed as a white, middle-class woman who remained at home full-time and found complete fulfillment in domestic life [23]. She is self-sacrificing, places everyone else’s needs above her own, is present, nurturing, patient, “moral”, and socially compliant, and conforms to the dominant norms of femininity and family [24]. A crucial aspect of implementing and sustaining this social identity is looking and acting the part; mothers feel the intense burden of performing motherhood flawlessly [24, 25]. Some conclude that this myth is deeply embedded in our lives, passed down from generation to generation, evolving to align with contemporary culture and society, yet always rooted in a fictitious notion of what constitutes the perfect mother [26]. Further, the “good mother” ideal now takes root even earlier, emerging in the form of the “good pregnant woman” and the “good birther”- adding new layers of pressure to an already demanding standard [27]. Research demonstrates that many mothers cannot conform to the “good NICU mother” ideal - always at the bedside [28], emotionally regulated, knowledgeable and comfortable with medical terminology [29], providing a plethora of breastmilk [30], and intuitively responsive to her infant’s needs. There are no structural barriers for the “good NICU mother” [28], no transportation or childcare challenges, no thoughts of returning to work due to unpaid leave [31], and certainly no thoughts of their own perinatal trauma [32].

While prior research has examined parental experiences of NICU settings, including barriers to engagement and the challenges of neonatal transport [33–35], less attention has been paid to how racialized narratives of Black motherhood shape maternal experiences during preterm birth and early NICU care. We propose that the prevailing ideologies of the “good NICU mother” intersect with historical, racist narratives that portray Black women as “bad mothers” [36, 37], heightening the risk for societal repercussions in the context of the NICU. This study examines how Black mothers navigate birth, neonatal transport, and the NICU hospitalization of their premature infant within these intersecting structural and institutional contexts.

Building on our previously adapted National Institute on Minority Health and Health Disparities (NIMHD) framework [38], which conceptualizes health inequities as operating across institutional and structural domains, we situate these experiences within broader systems of medical authority and structural racism. And through this lens, we argue that institutional systems of neonatal intensive care regulate and shape early maternal identity following preterm birth and functions as a space where legitimacy is interpreted and evaluated. This study makes a distinct contribution by tracing how disrespect and racialized maternal regulation unfold not only during labor and delivery, but across the full perinatal continuum — from emergency response through neonatal transport and NICU hospitalization. In doing so, it extends the RMC framework into a critically understudied setting and advances understanding of how structural racism and institutional norms collectively undermine equitable, dignified care for Black mothers and their infants.

## MATERIALS AND METHODS

The research in this manuscript was part of a larger study, the protocol for which has been described in detail elsewhere [39]. Research questions were guided by an adapted model of the NIMHD Research Framework [38] (see Table 1.). The model was adapted to fit this population of Black mothers experiencing PTB and address the influences of their experiences across domains from the individual to the health care system levels.

### Project Overview

The primary study used a descriptive, exploratory, multiple-case study design and a mixed methods approach to examine the impact of neonatal transport on Black very preterm (VPT) infants and their parents. Aim 1 focused on infant health outcomes using data extracted from electronic medical records. Aim 2 explored parent psychosocial health, social vulnerability, and experiences of socioeconomic and racial inequities through ten standardized surveys and in-depth parent interviews.

### Participants

Purposeful sampling was employed to select information-rich cases, ensuring the most effective use of limited resources to address the research questions [40]. The study was conducted among Black mothers who gave birth to an infant born preterm between 24 to 30 weeks' gestational age at a Level I or II hospital in South Carolina, which required transport to a higher level of care. All cases were recruited from one NICU serving as the perinatal regional center for 16 counties. Study activities were approved by the perinatal center’s Institutional Review Board (Prisma Health Institutional Review Board (#1999826–1). Enrollment started in May 2023 and ended in January 2024. All eligible mother-infant dyads who met the inclusion criteria were approached for study enrollment, provided informed consent, and participated in the study methods over the course of the infant’s NICU hospitalization. Mothers of multiple gestation, as well as fathers of preterms, were also invited to participate in the study. During the study enrollment period, potential dyads were limited, primarily due to a high number of maternal transfers instead of neonatal transport, which limited enrollment. Each participant received a $25 incentive for both survey and interview completion to compensate for the time and burden.

### Interview Process

Structured interview questions (see Table 2.) were designed by the investigators to collect in-depth insights into each mother’s experience. The interviews aimed to explore the participants’ unique experiences throughout the known pregnancy period and through the NICU journey in their own words. The questions were scripted, but participants were encouraged to elaborate and provide additional details. The interview ended with an open-ended question inviting participants to share anything further they felt was important. Verbal consent to digitally record the session was obtained at the start of each interview, and each interview was conducted one-on-one between the mother and the interviewer. The first author manually transcribed all interviews. To ensure methodological completeness and transparency the study adhered to the Standards for Reporting Qualitative Research (SRQR) guidelines [41].

### Data analysis and reflexivity

A reflexive approach utilizing Braun and Clarke’s [42, 43] six-phase process was employed to conduct the thematic analysis. This is an accessible and theoretically flexible approach to qualitative data analysis that facilitates the identification and interpretative analysis of patterns or themes in a given data set [42]. Reflexive thematic analysis is considered a reflection of the researcher’s active role in knowledge production and represents their interpretive analysis of the data and their patterns of meaning. Research teams use this collaborative approach when aiming to achieve richer interpretations instead of a consensus of a single “correct” answer [43]. Our personal experiences informed analyses, as three of the researchers were nurses with experience in and an intimate understanding of preterm delivery, delivery team experience, neonatal intensive care, and neonatal transport.

The research team brought extensive clinical and scholarly expertise in neonatal and maternal health to this study. KFW who led the project, has 19 years of neonatal experience including 9 years as a neonatal transport nurse. She obtained all consents and conducted all interviews. RMD is a qualitative methodologist with experience in health disparities research and a neonatal practice background and guided the analytic process. RBD has over 30 years of practice in NICU environments and transport and is a research expert in physiological pathways in preterm infants, data collection, and case study design. NSB is an epidemiologist specializing in maternal and child health, particularly in the context of racial/ethnic disparities. TMF has a multidisciplinary background in sociology, social work, and behavioral science with particular expertise in racial and socioeconomic inequities in Black women’s health. These professional backgrounds provided familiarity with the clinical environments described by participants and insight into structural inequities shaping maternal and neonatal outcomes. However, the team also recognized that longstanding involvement could risk normalizing institutional routines and privileging clinical perspectives. In addition, the interviewer’s professional identity may have influenced how participants framed their experiences, potentially shaping what was emphasized or withheld. Throughout analysis, the team engaged in iterative discussions and analytic memos to ensure that interpretations remined grounded in participants accounts [44]. Disciplinary perspectives within the team were leveraged to challenge consensus and expand the analytic possibilities.

Phase one: Data familiarization: Familiarization demands the listening, reading and re-reading of the entire dataset across the entire depth to become intimately familiar. Additional deep immersion was facilitated by the gathering of all data and manual transcription of data by the first author. Recordings were available to the entire team.

Phase two: Generating initial codes: Each interview was viewed line-by-line to generate a brief descriptive or interpretive code for pieces of information that are interesting or informative and may be of relevance to the research question. This initial coding was performed by KFW, RMD & RBD.

Phase three: Generating themes: After all interviews were coded, the focus shifted to the interpretation and patterned meanings across the dataset. Relevant contextual data from healthcare providers' notes were incorporated to illuminate institutional perspectives where appropriate. Rather than generating isolated thematic categories, the team examined how patterned meanings unfolded across the trajectory of the PTB experience.

Phase four: Reviewing: This phase required the research team to conduct a review of the themes in relation to the coded data items and the entire dataset [42, 45]. The team refined the organization of themes and examined how they interacted across three phases of care: (1) preterm labor and initial contact with the healthcare system, (2) PTB at a referral hospital followed by neonatal transport, and (3) the maternal postpartum period during infant’s NICU hospitalization. This structure reflected participants’ chronological narratives and highlighted how maternal evaluation developed across institutional encounters.

Phase five: Defining and naming themes: In this phase, the team identified which data items to use as extracts when writing up the results. In this deep analysis, extracts which made a compelling account of our arguments of maternal performativity and the construction of a good mother were chosen for use. Field notes were used to deepen interpretation and ensure coherence with the broader theoretical framework.

Phase six: Producing the report: Although the write-up of qualitative research was interwoven into the entire process of the analysis [42], this last phase was completion and final inspection. Data saturation was not a guiding principle; instead, the focus was on depth, richness, and reflexive engagement with the data. Findings are presented in a narrative format structured around the phases of the perinatal trajectory to remain aligned with participants lived experiences, while drawing attention to the institutional processes that shaped them.

## RESULTS

Demographic data were collected from all participants (n = 7) at the beginning of the study (six mothers and one father). Descriptive statistics were used to report sample characteristics. All mothers (n = 6) had completed high school, and one was a college graduate. They gave birth at 24 to 28 weeks of gestation, with a mean infant birthweight of 821 grams. Six interviews were completed by five Black mothers between the ages of 24 and 42. (One mother was interviewed twice, and one mother did not want to complete an interview.) The interviews lasted an average of 35 minutes, with five taking place during the infant’s NICU admission, and one after discharge home.

Findings are presented in context of participants' experiences through the trajectory of the PTB process, which allows for a nuanced exploration of how racism, implicit bias, and the cultural expectations surrounding the “good NICU mother” shaped and complicated their perinatal experiences. The PTB process is categorized into three distinct phases: (1) preterm labor and initial contact with the healthcare system, (2) PTB at a referral hospital followed by neonatal transport, and (3) the maternal postpartum period during infant’s NICU hospitalization. In presenting results, we have selected direct quotes which are used to illustrate key experiences these Black mothers navigated, presented either as block quotes or italicized within the text. To maintain confidentiality, all names used in this manuscript are pseudonyms. Quotes are lightly edited for readability and clarity (e.g., removal of disfluencies).

### Phase 1: Preterm Labor and Initial Contact with The Healthcare System

These Black women participants shared their early pregnancy experiences, during which they often felt they did not conform to societal expectations of looking pregnant or embodying the role of a “good pregnant woman”, and the frustration this caused them. Danielle, who received late prenatal care - having attended only one prenatal visit before delivering, and prior to the first ultrasound - expressed that she had distanced herself from her friends, fearing judgment for having given birth to preterm twins.

People can curse you and not mean to. Just say, Oh, that girl don't need no more kids. You know what I'm saying? So I didn't really tell anybody and it was a shock… ‘didn't I see you last month? You weren't pregnant, didn't I see you two months ago and you weren't pregnant. Oh, I see you last week or you ain't look pregnant’.

She went on to describe how ridicule from others questioning her credibility as a mother compounded the emotional toll of her pregnancy, and deepened her sense of isolation and mistrust:

People don't understand a lot. All them kids you had you ain't know you was pregnant? What, you think Imma lie? I'm pretty sure I would tell you if I was… I hate when people say that. Girl, I just had literally six pregnancies. I should know, if I'm telling you I didn't (laughing) I really didn't. And I've had people literally ridicule me, laugh in my face type stuff…almost like I did it on purpose or something…that makes it harder to even want to share with people or talk to people about anything.

Disbelief extended beyond the social circle. During her prenatal visit, she recalled how her provider reacted when her baby moved:

When she did check me, my baby kicked her and she literally snatched her hand out my vagina because she said it was so “weird”. She said, “I'm used to breech babies, but that one caught me off guard” like my baby kicked too much.

Moments like this highlighted how a mother’s body experience can be devalued and “othered” through healthcare provider language and actions [46]; the reaction by this provider to a normal bodily process reinforced for this mother that her pregnancy, and by extension, her motherhood, was outside of what others consider normal.

Institutional discourse can reinforce social narratives that question Black women’s maternal knowledge and responsibility. This was mirrored in the medical record, where the social worker questioned the mother’s veracity regarding her explanation as to why she had only had one prenatal care (PNC) visit:

PNC visit x1, “reportedly” secondary to lack of awareness of pregnant state. [emphasis added]

Clinical encounters reveal how implicit bias intertwines with gendered expectations of the “good mother,” where empathy and respect are often granted only to those perceived as compliant or well-informed. These judgments permeate the perinatal experience, shaping how mothers come to perceive themselves as inadequate or unprepared. Jasmine, a second time mother, recalled:

So when I had… my first daughter, that one nurse that treated me like crap and asked me ‘did I not go to Lamaze class?’ and I need to listen to how they told me to breathe.

Imani, a 24-week pregnant mother experiencing contractions every 2 to 3 minutes called 911 for transport. She was panicking when the Emergency Medical Service (EMS) providers arrived and told them:

‘I think I'm in labor!’ So, they walked me down the stairs and I got in the back of the ambulance, and they was asking me like a few questions… So once we started pulling off, like once we left out of my apartment complex, we wasn't even out of [the city] yet, and I notice, like the contractions was like 2 or 3 minutes apart. And I had a further drive to go because [the local hospital] is like thirty minutes away from where I live… the doctor [at the local hospital had] told me to go to, come straight to [the regional referral center] ….cause [the local hospital] didn't have a NICU. So, if anything went wrong, and I had to deliver right then.. they didn't have the tools they needed for the baby once he came out.. So I said, okay, but… [the EMS drivers] asked me ‘do you want to go to [the local hospital] first since your doctor is down there, and then see if they [want to] transport you if everything not looking too good?’ And I said Well, yeah, that's fine.

Despite reporting active labor at 24 weeks, the EMS workers did not place her on a stretcher before loading her into the ambulance nor did they check her labor progress.

Latoya went into labor at 26 weeks’ gestation at home and felt the urge to push while she was on the toilet. Having been told that her infant was in a breech position, she called EMS for assistance:

The ambulance had pissed me off so bad because they didn't take my situation as emergent. They put me as a non-emergent because they didn't see my baby between my legs. So, they drove to the hospital at normal speed going through the lights, highway. And when I say they made me so furious, it made me so furious because she didn't even examine me. She just had me open my legs and just left it like that. But I'm telling her like, I never felt like this before, you know, this is my fifth baby - I would know.

### Phase 2: PTB at a Referral Hospital and Neonatal Transport

When asked about their experiences with PTB, several participants reported instances in which they felt disregarded or undervalued by various healthcare providers at local healthcare facilities. One mother described how she felt an absence of awareness and empathy and even physically mishandled by a healthcare provider:

Danielle described *There was a nurse that really made me want to strangle her. She literally was the most unaware individual I've ever met. She personally made the delivery for the twins hard…my belly and her belly were both big- and she was short, so trying to get the straps on me…this lady laid into my already hurting belly…she did it several times and the third time I caught her under her titty, I didn't care and pushed her upwards. Like you don't feel me under here? Like you don't feel that you're on top of me and you're putting your weight down on something that's already going on. That hurt! And she looked at me like, ‘oh, you know’, just kind of waved me off*.

Imani (24-weeks pregnant) arrived at the labor and delivery floor via EMS. She had been in labor for several hours and repeatedly expressed that she was preparing to deliver the baby. She stated:

The doctor came in there. And she was asking me [questions like] how long I have been in pain, and, what it felt like, and I couldn't even talk to them I was just getting so frustrated because I was in so much pain, and I started screaming please help me! Like, there's no way I can keep talking.

Instead of checking her labor progress, the clinicians arranged for maternal transfer without knowing if it was an imminent birth. The maternal transport would have taken over two hours. Imani went on to add:

There's nothing I can do from stop pushing cause I felt the baby coming…there was nothing I could do. They was talking to me, tell[ing] me, ‘no, [you are] just pushing because [you] think [you are] in so much pain’. So, when they looked down again, I told them, I was like, I felt the head coming out.

Danielle went into labor at home. Upon her arrival at the hospital, she was taken to the labor and delivery surgical suite for a cesarean section. She stated:

The doctor was talking about my situation all over me, but not explaining anything to me, not telling me what was going on around me …. [if he had] it would have probably put me at ease. None of that was going on. It was almost like I was a dang guinea pig, like a little lab rat. And they were discussing what they were going to do to the little guinea pig next.

Mothers with an infant in the NICU often experience a profound sense of loss of control, coupled with feelings of helplessness and exclusion [29, 47]. These emotions are intensified when an infant is transferred to another hospital, frequently leaving mothers feeling like outsiders. Jasmine expressed uncertainty about whether her infant would even survive the transport:

They didn't call me [to] let me know that she made it. I actually had to call. I had to call them, I got the hospital phone in my room and say, hey, has my baby made it? Have you guys heard anything?

Danielle described waking up from her cesarean section confused, alone, and still in pain:

When I woke up, I woke up in so much pain. I was crying, begging this lady, can I please, you know, are y’all going to give me something like, can y'all you put me back to sleep or like because- man, when I say they ignored me … there's nobody there but one of the nurse[s] and …well of course, you know- recovery is very sterile looking. It's nothing but grey walls. And I just felt so alone. I was panicking. And the lady that was in the corner just putting in stuff in the computer I could barely see. I'm literally having to look kind of like back and over to even notice that she's there. She just kind of waving me off, well, you're fine. I'm sorry, honey, there's nothing we can do for you until such and such and such happens.

She goes on to talk about how pain and isolation gave way to a deeper need for human connection:

At least wheel me to my room so I can see my mom or somebody that love[s] me. You know what I'm saying? That can kind of talk me through it. But it was agonizing. Like I never felt- again I done did this before- a C-section and several babies it's supposed to hurt, Okay? That was my decision to have babies. Blah, blah, blah, blah, blah. But I've never felt this! And I'm still in pain.

Brianna, a 28-year-old first-time mother, was finally being discharged from the maternity hospital after three days. She had never seen her infant but had been informed of the NICU visitation hours:

The day that I was supposed to be discharged, they was supposed to be discharging me early so I could go in the hospital to see her. But they discharged me so late that by the time I got done, the visiting hours [in the NICU] was over, so I had to go home. I had to drive [the whole one hour] home. Well, my fiancé had to take me all the way home. Then he had to come back up here, [the next morning get me, then drive another hour] back up there to the NICU.

She went yet another day without seeing her infant and, after having a cesarean section, she spent an additional two hours in the car. No one ever told Brianna “*this is your baby; of course, you can come”*, or that an exception to visiting hours could be made. No one empowered her to embrace her role as a mother. Jasmine was left feeling as though her body had failed her:

We already feel like failures because we wasn't able to carry our baby full term…I have no control over our child. My child was taken from me [at] 25 weeks and five days. you took my child from me, my bonding with her.

### Phase 3: Postpartum and the NICU

Experiences of powerlessness did not cease after childbirth; rather, they persisted throughout the entire duration of the NICU stay. Jasmine emphasized the loss of parental identity, feeling reduced to a mere passive observer, stating:

I felt like that nurse just took my role as a mother, like she was making decisions for my child instead of me. Like I had to ask to touch my child… this was 45 days [after birth] when I was able to finally touch my child.

After 60 minutes of skin-to-skin with her infant, Jasmine was told her time limit was up and to place the infant back in the incubator- leaving her powerlessness in her role as a mother:

I said wait a minute. No one's told me that. The first time I held her the nurse let me hold her all day long. She said ‘well her body temperature, you could be hurting your baby when you’re doing that’. And I was like, Are you serious?… She took my baby from me and I took a video of it.. it was the first time I heard [her] cry… It was like a faint cry, and that broke my heart to pieces. So I said, Can I at least touch her? She said, ‘That's fine as long as you don't touch her heart monitor’. So I held her hand. I stood there. Me still healing, blood pressure, getting light-headed, I stood up because no one offered to even lower the bed for me so I could sit down and hand hug her. So, I stood up for two hours… I wouldn't put my child in harm's way. This is my child. I would have died for her. So, to pull her from my chest, just because you think her being on my chest is going to hurt her? That's just crazy to me.

For this mother, the loss of physical connection became a deeper loss of control and loss of identity. Jasmine continued:

I felt powerless. That's another feeling that's a weird feeling. Like this is my child, this child that was inside my body for six months and this is my body. She left my body way before it was time. She should still be in my body right now. And now that she's outside my body, you guys are not even letting me touch or feel or be around my child. I'm her mother, I'm the one that brought her into this world and you're taking her from me. I felt like that nurse just took my role as a mother, like she was making decisions for my child instead of me. Like I had to ask to touch my child- [to] have to ask, like I felt like that nurse just took my position as her guardian and as her protector.

Latoya talked to us about her depression:

I wasn't really in a happy state…I try to hug on my kids as much as I can to light me up. It's really just my husband [that I have for support], if not my husband, then I try to uplift myself sometimes. I try not to really too much think about it.

When we asked if she had spoken with her doctor about her depression, she replied *“I did, but she didn't really say too much.”*

### Repercussions

The powerful emotions of guilt and inadequacy were magnified by the labels assigned by NICU healthcare providers. “Bad mother” speculation from providers only deepened their emotional turmoil. For example, a Department of Social Services case was initiated on Imani, who was caring for her four young children at home, after the social worker documented in the chart that the:

NNP expressed concerns regarding mother of baby’s poor visitation and difficulty in making contact.

Jasmine spoke of the anguish caused by a phone call from the lactation consultant, who said:

“I see that you haven't a whole lot of luck with milk. Okay, well, we're going to need that breast pump [returned] we had influx of babies and you probably don't need it as much as them”. But that was like so insulting. So insulting. I'm suffering from anxiety attacks. They just don't know half of what I'm battling with. And for you to um, make me feel like I'm failing because I'm not producing enough milk! But to me, that was just so insensitive.

### Affirming motherhood

Across the three identified phases, we were also informed of instances when performativity was reinforced in subtle ways. The transport team wheeled the incubator to Danielle’s room so she could see her infant. A team member assisted her in sitting up in bed and reaching through the portholes to touch her baby. She stated:

The nurses did not move to try to help me, but that transport guy grabbed me by my arm and under my right shoulder. And he said, well, if you gonna f* yourself up, let's f* yourself up together, you know what I'm saying? Let's get you where you need to be to make you feel better. And he literally- gently too, like he didn't force me - every bit of effort I was giving, he just allowed another like arm to have. Like he let me rest that weight on him. I appreciate him more than anything. I don't know his name. I wish I did. I wish I could shake his hand right now. He made me feel like I mattered, you know, to them. And he wanted to help me do something to make me feel better.

That sense of affirmation was occasionally encouraged by the simple gesture of being called mama:

Jasmine told us: *[That nurse] was awesome. She took a picture of us. She said, Mama, you hold your baby as much as you can*.

Emotional recognition provided significant support during challenging times. She went on to say:

You know those that are positive moments, those positive moments where the nurse held [and] hugged me after that [other] nurse took my baby from me. She hugged me. And she's like I'm so sorry you had to go through that.

## DISCUSSION

This study is the first to examine how Black mothers experienced birth, neonatal transport and early NICU hospitalization within institutional systems of neonatal intensive care. Rather than sole instances of bias or miscommunication, our findings suggest that Perinatal systems of care- including emergency response, labor and delivery units, neonatal transport, and the NICU- collectively function as institutional sites in which maternal identity is interpreted and evaluated. Across these phases, participants describe dismissal, constrained communication, limited agency, and scrutiny. These interactions reflect more than isolated negative experiences; they illustrate how institutional norms and racialized assumptions shape whose maternal authority is recognized and whose is questioned.

The construct of the “good NICU mother” emerged as a regulatory framework within this broader system of care. Expectations of constant presence, emotional composure, fluency in medical jargon, and uninterrupted breastmilk positioned maternal legitimacy as contingent upon meeting institutionally defined standards. Importantly, these expectations did not originate solely within the NICU but were introduced earlier in pregnancy and labor through interactions that called into question maternal knowledge and credibility. For Black mothers navigating longstanding narratives of suspicion and diminished competence, these expectations carried particular weight. In this way, the NICU operated not only as a site of life-saving neonatal care, but intensified and formalized processes of maternal evaluation already set in motion across prior institutional encounters.

These experiences, read through the lens of respectful maternity care, reveal a systematic failure to uphold the conditions the WHO defines as fundamental rights. Participants were not afforded dignity in their clinical encounters- their concerns were dismissed, their knowledge subordinated, and their behaviors surveilled. They were not free from mistreatment; they described being ignored during labor, separated from their infants without adequate explanation, and subjected to social services referrals that carried implicit moral judgments. Nor were they empowered to make informed choices. Limited communication, medical jargon, and constrained agency across the perinatal continuum reduced their capacity to advocate for themselves and their infants. Taken together, these accounts suggest that disrespect in maternity care does not end at delivery; for Black mothers of preterm infants, it extends and, in some cases, intensifies across the NICU hospitalization.

Consistent with our adapted NIMHD framework, these findings illustrate how inequities operate across interconnected structural, institutional, and interpersonal domains. At the interpersonal level, provider dismissal and limited communication during pregnancy and labor undermined maternal credibility. At the institutional level, documentation practices, visitation policies and lactation monitoring formalized expectations of maternal behavior. At the structural level, racialized narratives of Black motherhood shaped how maternal actions were interpreted across these settings. Together, these layers demonstrate how maternal identity is produced through cumulative institutional processes rather than within a single clinical site.

Our findings align with prior research documenting the emotional disruption, loss of control, and identity challenges experienced by parents of NICU infants [32, 34]. Like studies identifying structural barriers to parental presence and engagement [28, 33], participants described transportation barriers, competing caregiving responsibilities, and institutional rules that limited bedside time. However, our data extend this literature by demonstrating how limited presence may be interpreted within a moral framework. Absence or reduced visitation was not merely a logistical challenge, it was sometimes read as insufficient maternal commitment, reinforcing institutional hierarchies of legitimacy.

Similarly, our findings corroborate research on communication breakdowns and limited parental voice in neonatal settings [29, 48]. Prior work emphasizes how inadequate communication increases stress and reduces parental engagement. Our study suggests that communication practices also function as mechanisms of credibility. When mothers’ concerns were minimized, reframed or documented with skepticism, maternal knowledge was subordinated to institutional authority. In this sense, communication operated not only as a relational process but as a site where maternal legitimacy was negotiated.

Extensive scholarship has documented racial disparities in maternal morbidity, mortality, and neonatal outcomes [15, 18], as well as historical stereotypes portraying Black women as irresponsible or neglectful mothers [36, 37]. Yet few qualitative studies trace how these racialized narratives are enacted across everyday perinatal care interactions. By linking historical patterns of diminished credibility to moments of emergency response, labor triage, social services referral, and NICU bedside regulation, this study fills an important gap. Our findings suggest that institutional expectations of the “good NICU mother” are not neutral standards of engagement; they intersect with racialized assumptions about responsibility and competence, shaping how maternal behaviors are interpreted and sanctioned.

Drawing on Butler’s conceptualization of performativity, motherhood across perinatal care settings can be understood as an identity enacted under observation. Maternal legitimacy was not presumed but continually assessed through visible behaviors- attendance, affect, milk production, phone call responsiveness. When performance aligned with institutional expectations, mothers were affirmed. When it deviated, maternal authority was questioned and scrutinized. For Black mothers, whose maternal competence has historically been contested in public and medical discourse, this cumulative environment may intensify vulnerability to surveillance.

These findings have direct implications for efforts to advance respectful maternity care and address inequities across the full perinatal continuum. The WHO’s RMC framework has historically centered labor and delivery, yet our findings make clear that disrespect, mistreatment, and constrained agency persist well beyond the delivery room. Extending RMC accountability into neonatal intensive care settings, where maternal legitimacy is continually evaluated, is essential for achieving equitable, dignity-centered care. Interventions focused solely on individual bias training may be insufficient if institutional routines, documentation practices, visitation policies, and criteria for social service referral continue to encode narrow definitions of maternal engagement. Structural approaches may include re-evaluating policies that implicitly equate presence with competence or caring, strengthening accountability mechanisms around documentation of maternal concerns, and embedding culturally responsive family-centered care models across the entire perinatal continuum. Addressing disparities requires examining how institutional definitions of maternal legitimacy are constructed, operationalized and reinforced across systems of care. Our findings encourage a reevaluation of the good mother label in the NICU, acknowledging the complexities of the various roles that parents are performing.

Several limitations of this study warrant consideration. The sample was small and drawn from a single perinatal region in the Southeastern United States, which limits generalizability; experiences may differ across other geographic and institutional contexts. Although all six eligible mothers were recruited during the enrollment period, one declined to participate. Additionally, while the research team brought substantial clinical expertise in neonatal and transport care, this positionality may have shaped interpretation of participants’ accounts. Reflexive practices were employed throughout analysis to critically examine these assumptions.

## CONCLUSION

This study demonstrates that the transition to motherhood following preterm birth unfolds within interconnected perinatal systems of care that do more than deliver clinical services. Across emergency response, labor and delivery, neonatal transport, and NICU hospitalization, maternal identity was interrupted, evaluated, and at times, constrained. By situating Black mothers’ accounts within our adapted NIMHD framework, we show how racialized narratives of credibility and responsibility operate across structural, institutional, and interpersonal domains to shape maternal legitimacy during moments of crisis.

The “good NICU mother” emerges not simply as a cultural ideal, but as an institutionalized standard against which maternal behavior is assessed one that, for Black mothers, operates in direct tension with the principles of respectful maternity care. Recognizing these processes invites a broader reconsideration of how maternal competence is defined, measured, and supported across perinatal care environments. Achieving RMC in its fullest sense requires extending its principles beyond the delivery room and into neonatal intensive care, where dignity, freedom from mistreatment, and informed choice remain equally fundamental. Advancing equity in maternal and neonatal health requires attention not only to clinical outcomes, but to the institutional practices through which motherhood itself is regulated. Future research should further explore the short- and long-term psychological, social, and identity-based impacts of PTB and neonatal transport on Black mothers and examine how RMC frameworks can be operationalized across the full perinatal continuum to support more inclusive, equitable definitions of what it means to be a “good NICU mother”.

## Figures and Tables

**Table 1:** Relevant theoretical constructs from framework

INDIVIDUAL LEVEL	INTERPERSONAL LEVEL	COMMUNITY LEVEL	SOCIETAL LEVEL
**Psychological Stress, Anxiety** (Fear, loss of control, powerlessness, loss of identity.) BEHAVIORAL DOMAIN	**Patient–Provider Communication** (Clarity, empathy, and cultural sensitivity of providers during critical moments like birth and transfer.) HEALTHCARE SYSTEM DOMAIN	**Hospital Resources** (Lack of NICU leading to transport & separation of mother and infant, geographic location determines access.) PHYSICAL/BUILT ENVIRONMENT DOMAIN	**Societal Norms & Values** (Normative expectations of strength and endurance.) BEHAVIORAL DOMAIN
**Medical Mistrust** (Past or present experiences of racism in healthcare may shape perceptions of care quality and safety.) SOCIOCULTURAL DOMAIN	**Social Support** (Emotional support from family, friends, or community members.) SOCIOCULTURAL DOMAIN	**Access to Transportation** (Whether families can visit their infant post-transport or access support at receiving hospitals.) HEALTHCARE SYSTEM DOMAIN	**Policy Environment** (Influence of transfer protocols, Medicaid coverage, or regional NICU access on care pathways.) HEALTHCARE SYSTEM DOMAIN

**Table 2:** Sample interview question

Baseline interview question	Sample Probe	Theoretical Constructs Addressed
Tell me about your pregnancy experience over the last_months and what really stands out to you about this time in your life.	Who is the person or people you feel gave you emotional support during your pregnancy?	Individual beliefs regarding medical care Patient-Clinician relationship Coping Strategies Social support at the behavioral level Experiences of racism or discrimination

## Data Availability

The qualitative data generated and analyzed during this study are not publicly available due to the sensitive nature of the information shared by participants and to protect participant confidentiality. Deidentified data may be made available from the corresponding author on reasonable request.

## Abbreviations

NICU: neonatal intensive care unit
RMC: respectful maternity care
NIMHD: National Institute on Minority Health and Health Disparities
PTB: preterm birth
SRQR: Standards for Reporting Qualitative Research
